# The State Board of Health

**Published:** 1877-01-20

**Authors:** 


### THE STATE BOARD OF HEALTH

The Georgia State Board of Health assembled in Atlanta on the 17th inst. A report was presented by Dr. J. P. Logan and the State Geologist, embodying the facts and conclusions of the late investigations made by the Board, at Savannah, touching the yellow fever epidemic. A report was also made by Prof. H. T. Campbell, of Augusta.

The Board alluded in severe terms to the imperfect sanitary condition of Savannah previous to the outbreak of the fever. Malarial influences were unusually prevalent, and the meteorological conditions were unfavorable—the weather being unusually warm, and the rain-fall unprecedentedly heavy.

The Board, however, does not attribute the origin of the fever to local causes, but gave facts to show that the germs of the disease at Savannah, Brunswick, and Doboy were unquestionably imported from the West Indies in Spanish vessels.

The conclusions of the Board upon this point, we learn, are not concurred in by many of the resident physicians of Savannah.

If, when we see the published transactions of the Board, there shall appear anything important, or previously unknown to the profession, we will give it to our readers.

